# Global Status of *Toxoplasma gondii* Seroprevalence in Rodents: A Systematic Review and Meta-Analysis

**DOI:** 10.3389/fvets.2020.00461

**Published:** 2020-07-31

**Authors:** Tahereh Mikaeili Galeh, Shahabeddin Sarvi, Mahbobeh Montazeri, Mahmood Moosazadeh, Maryam Nakhaei, Seyyed Ali Shariatzadeh, Ahmad Daryani

**Affiliations:** ^1^Department of Parasitology, School of Medicine, Mazandaran University of Medical Sciences, Sari, Iran; ^2^Toxoplasmosis Research Center, Mazandaran University of Medical Sciences, Sari, Iran; ^3^Student Research Committee, Mazandaran University of Medical Sciences, Sari, Iran; ^4^Fatemeh Zahra Hospital, Mazandaran University of Medical Sciences and Health Services, Sari, Iran; ^5^Health Sciences Research Center, Addiction Institute, Mazandaran University of Medical Sciences, Sari, Iran

**Keywords:** toxoplasmosis, *Toxoplasma gondii*, seroprevalence, rodents, systematic review, meta-analysis

## Abstract

Toxoplasmosis is one of the most prevalent infections in humans and animals caused by the intracellular protozoan parasite *Toxoplasma gondii* (*T. gondii*). Rodents, as intermediate and reservoir hosts, play a key role in the maintenance and transmission of *T. gondii*. They can be contaminated and maintain the parasite in the form of cysts in their bodies, demonstrating an infection source for their offsprings, predators (particularly felids), and other animals. Therefore, the present systematic review and meta-analysis study was carried out to evaluate the global seroprevalence of *T. gondii* in these mammals. For achieving the purpose of the current study, six English databases (PubMed, Science Direct, Web of Science, Scopus, ProQuest, and Google Scholar) were systematically searched for related studies from 1970 to 2018. Finally, a total of 52,372 records were screened, 105 records including 26,221 rodents were incorporated in the present study. By random effect models, the overall seroprevalence was calculated at 6% (95% CI = 6–7%), with the highest amount was observed in Africa (24%) and South America (18%), and the lowest amount in Europe (1%). The subgroup data analysis by gender manifested that the prevalence of Immunoglobulin G antibodies did not differ between genders (*P* > 0.05). Due to the significant heterogeneity, meta-regression models were applied based on serological techniques and continental regions; however, the obtained values were not statistically significant (*P* = 0.480 and *P* = 0.295, respectively). The present study revealed a relatively low level of *T. gondii* seroprevalence in rodents; however, if they were the main food source for their predators, they would cause high transmission of *T. gondii*.

## Introduction

Toxoplasmosis is a highly prevalent zoonotic parasitic infection caused by *Toxoplasma gondii* (*T. gondii*), an obligate intracellular apicomplexan protozoan, that infects nearly 30% of the world human population (1, 2). This foodborne pathogen has complex life cycles, including the sylvatic transmission cycle in forest habitats, and domestic transmission cycle in human settlements, which might be hardly connected (3).

Felids as definitive hosts, excrete oocysts through feces (sexual stage) which infect intermediate hosts, a large range of homoeothermic animals (e.g., rodents and humans), resulting in the formation of tissue cysts (asexual stage) (4, 5). Transmission to intermediate hosts can also occur via two other main ways of congenitally or by eating undercooked meat containing tissue cysts (3, 6).

*T. gondii* in immunocompetent people is mostly asymptomatic, or with non-specific flu-like symptoms. However, this single-celled microorganism is medically important and causes serious consequences in immunocompromised people and pregnant women (7). The life-threatening encephalitis can occur in immunocompromised humans following the infection (5, 8). Primary infection during pregnancy can result in congenital toxoplasmosis with abortion, neonatal death, chorioretinitis, and neurological disorders in the unborn child (9, 10).

Rodents, the largest order of the class Mammalia with a number higher than the total number of other mammals, are characterized by upper and lower pairs of ever-growing incisors and a set of chewing teeth. They have short reproductive cycle and high compatibility for living in various habitats (11). They are responsible for the zoonotic transmission of several diseases to humans. Rodents play an important role in the maintenance of the *T. gondii* life cycle and epidemiology of toxoplasmosis because they are considered as reservoirs and carriers of the disease and the main source of infection for cats and their relatives (12, 13). This role is more important in species that live close to human habitats, because of the importance of its environment and human health. Establishing the infection transmission cycle by rodents causes releasing oocysts from infected felids and the spread of contamination in the environment, and thus increasing the infection risk of each of the parasite hosts in the environment, most importantly of humans in its habitats (14).

Direct transmission of toxoplasmosis from rodents to humans may occur when they are consumed as food by humans, as it is done by many human populations. For example, rodents such as rats and capybaras (*Hydrochoerus hydrochaeris*), one of the largest rodents in the world, are used by some nations and may be a source of *T. gondii* if their meat containing parasitic cysts is consumed undercooked (3, 15, 16). Therefore, it is necessary to pay attention to hygienic principles when preparing and cooking rodents in such populations. Furthermore, if rodents are accidentally eaten by livestock, they could mediate disease transmission to humans (11).

Considering the rodents' importance in the transmission of toxoplasmosis to felids and humans, as well as, abundance and distribution of rodents near the human settlements and in absence of a comprehensive study, we performed a global meta-analysis to assess the pooled seroprevalence of *T. gondii* in this mammals.

## Methods

### Design and Protocol Registration

This extensive research was conducted in accordance with the items reported in the PRISMA statement (www.prisma-statement.org). The details of the study protocol are available on the website of the International Prospective Register of Systematic Reviews with the identifier Central Registration Depository of 42018107622 (17).

### Search Strategy

To elucidate the seroepidemiological status of *T. gondii* in rodents, an extensive and principled search was carried out on scientific publications from 1970 to 2018 using six English language databases of the following websites: (www.pubmed.gov), (www.sciencedirect.com), (www.webofknowledge.com), (www.scopus.com), (www.search.proquest.com), and (www.scholar.google.com).

The keywords were used based on medical subject heading terms: “*Toxoplasma*,” “Toxoplasmosis,” “*T. gondii*,” “Seroprevalence,” “Seroepidemiology,” “Prevalence,” and “Rodentia.” In addition, perusing the reference lists to retrieve additional related publications was conducted manually.

### Study Selection

For the purpose of eligible screening, all the retrieved titles, abstracts, and full-texts if needed, were carefully perused and eligible studies were selected by two independent authors (TMG and MM). Disagreements, if any, were discussed and sorted out by consensus.

Finally, studies with full texts or abstracts available in English which examined the seroprevalence of antibodies against *T. gondii* in rodents with the total sample size larger than 20 were selected. The reviews, experimental, human-based, non-serological, repetitive manuscripts and those with inadequate data were excluded from the present study.

### Data Extraction and Quality Assessment

The data extraction process was performed by two independent authors (MM and TMG) and disagreements were resolved by discussion and consensus. Using an information extraction sheet, the following data were recorded from the selected studies: first author, publication year, geographical region, sampling period, total sample size, gender and age distribution, number and percentage of seropositive rodents, and serological methods. The quality of included records was appraised using the Joanna Briggs Institute (JBI) Prevalence Critical Appraisal Tool (18).

### Statistical Analysis

The present meta-analysis was carried out using Stata software (version 15; Stata Corp, College Station, TX, USA). Point estimations and 95% confidence intervals (CI) of anti-*Toxoplasma* Immunoglobulin G (IgG) seroprevalence were calculated for all the selected records. Chi-squared and I-squared tests were applied to evaluate the extent of variations among the independent studies. The I-squared values of lower than 25%, 25–50%, and higher than 50% were considered as low, moderate, and high heterogeneity, respectively.

To explore the causes of heterogeneity among the selected studies, meta-regression and subgroup analysis were performed based on serological techniques and continental regions. The subgroup analysis was also conducted according to the genders. The publication bias was examined by Egger's regression test and funnel plot asymmetry. According to the results of the heterogeneity test, a random effect model was used to pool the estimates and a forest plot was drawn to visualize the outcomes.

Furthermore, to evaluate the effect of each study on the overall effect size, a sensitivity analysis was performed by eliminating a single study at a time.

## Results

In this universal scientific research, initially 52,372 records were retrieved through principled search, 105 records from 44 countries were finally appraised appropriately to be entered into this global research. Totally, 26,221 rodents and 2,263 positive cases were analyzed for IgG antibodies against *T. gondii*.

**Figure 1:**
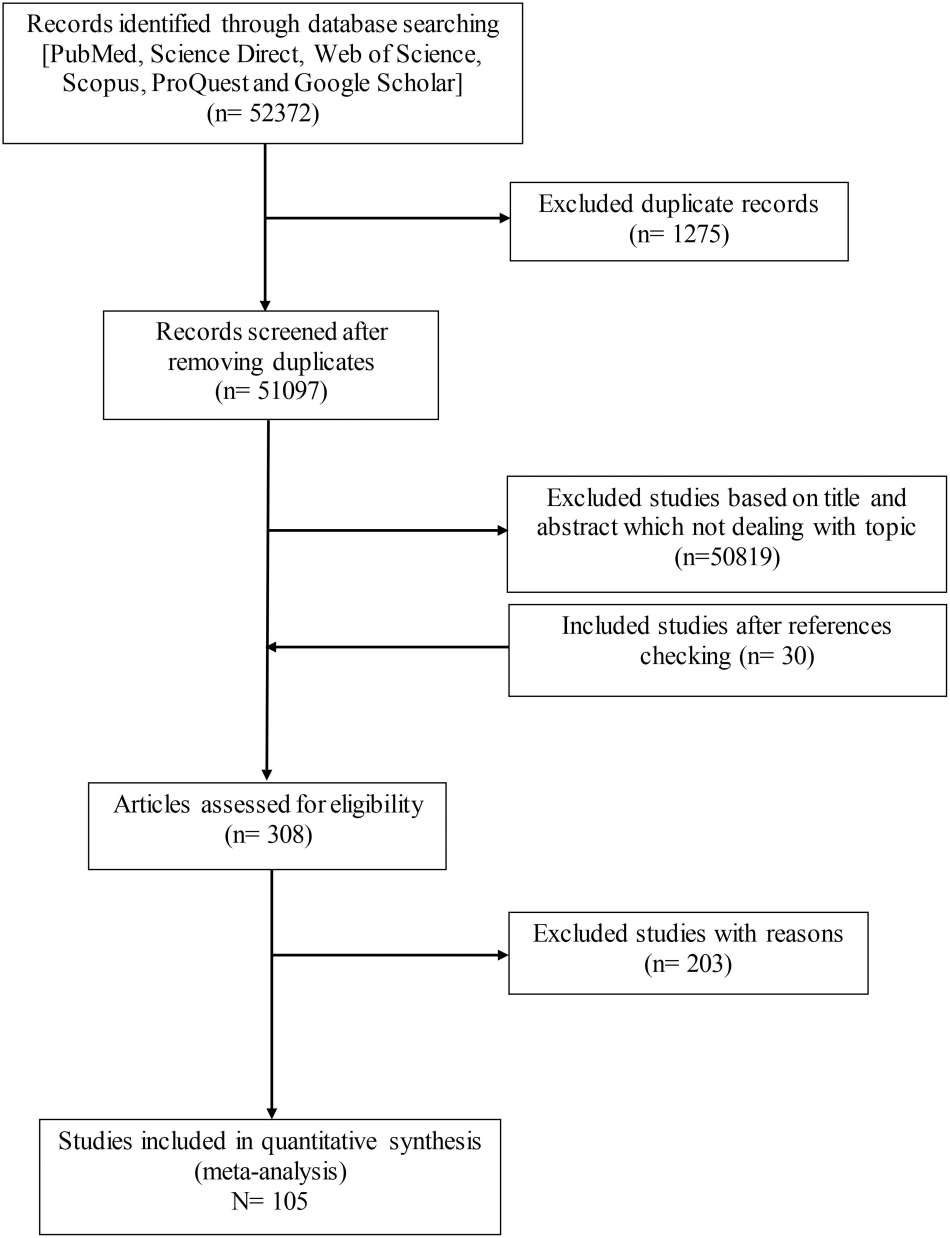
The PRISMA flow diagram describing the study design process.

Details of the search and study selection procedure are described in a PRISMA flow diagram (Figure 1). Table 1 lists the basic characteristics of the selected papers. A study conducted by Dabritz et al. (13), had two datasets (13) and only two studies were available for the continent of Australia (52, 53). The most performed serologic tests in the literature were, including modified agglutination test (MAT), Sabin-Feldman dye test (SFDT), indirect fluorescent antibody test (IFAT), latex agglutination test (LAT), direct agglutination test (DAT), enzyme-linked immunosorbent assay (ELISA), and indirect hemagglutination test (IHAT) in 39, 17, 14, 9, 7, 6, and 5 studies, respectively. The other serological tests were conducted in nine studies. The average score obtained from the JBI scale was six illustrating the moderate to the high quality of the selected records (Table 1).

**Table 1:** Baseline characteristics of selected studies reporting seroprevalence of *T. gondii* in rodents.

**Continent/ References**	**Country**	**Sample size**	**IgG Seroprevalence (%)**	**Serological method**	**Cut off**	**Quality score**
**Africa**						
(19)	Egypt	100	34 (34)	SFDT	≥1:16	6
(20)	African countries	235	21 (8.9)	SFDT	≥1:40	6
(21)	Egypt	110	47 (42.7)	SFDT	–	6
(22)	Nigeria	104	104 (100)	SFDT	–	6
(23)	South Africa	217	9 (4.15)	LAT	–	6
(14)	Niger	765	15 (1.96)	MAT	≥1:16	6
(24)	Canary Islands and Cape Verde	185	22 (11.89)	IFAT	–	6
(25)	South Africa	137	15 (10.95)	LAT	–	6
(26)	Senegal	1,205	44 (3.65)	MAT	≥1:16	6
**Asia**						
(27)	Taiwan	29	0	IHAT or SFDT	–	5
(28)	Georgia	44	0	SFDT	≥1:4	5
(29)	Japan	245	64 (26.12)	LAT	≥1:4	6
(30)	Georgia	31	20 (64.52)	IFAT	≥ 1:32	5
(31)	India	186	18 (9.68)	IHAT	–	6
(32)	Japan	65	0	LAT	≥1:64	5
(33)	China	955	9 (0.94)	IHAT	≥1:64	6
(34)	South Korea	1,008	15 (1.49)	ELISA	–	6
(35)	Saudi Arabia	25	5 (20)	IHAT	–	5
(36)	Iran	90	0	IFAT	–	6
(37)	Turkey	105	12 (11.43)	SFDT	≥1:16	6
(38)	China	124	36 (29.03)	MAT	≥1:20	6
(39)	Philippines	157	53 (33.76)	DAT	≥1:256	6
(40)	Israel	27	21 (77.78)	MAT	≥1:16	5
(41)	China	217	7 (3.23)	MAT	≥1:40	6
(3)	Thailand	461	21 (4.6)	LAT	≥1:64	6
(42)	Iran	150	55 (36.67)	ELISA	–	6
(43)	Iran	127	31 (24.41)	ICT	–	7
(44)	Pakistan	300	156 (52)	LAT	>1:16	6
(45)	South Korea	625	15 (2.4)	ELISA	–	6
(46)	Malaysia	526	19 (3.61)	IFAT	≥1:64	6
(47)	Thailand	60	3 (5)	ILAT	≥ 1:64	5
(48)	Russia	257	8 (3.11)	EIA	–	8
(49)	Iran	52	3 (5.77)	MAT	≥1:40	5
(50)	Pakistan	112	47 (41.96)	LAT	≥1:16	6
(51)	China	261	32 (12.26)	MAT	≥1:20	6
**Australia**						
(52)	FSM (Namoluk atoll)	658	50 (7.6)	SFDT	≥1:8	6
(53)	Queensland	179	3 (1.67)	CFT	≥1:8	6
**Europe**						
(54)	Austria	109	0	SFDT	–	6
(55)	France	1,175	7 (0.59)	SFDT	–	6
(56)	Norway and Sweden	732	3 (0.41)	SFDT	≥1:8	6
(57)	Scotland	125	20 (16)	SFDT	≥1:10	6
(58)	Scotland	106	1 (0.94)	SFDT	≥1:10	6
(59)	UK	235	84 (35.74)	ILAT	>1:10	9
(60)	Italy	41	15 (36.58)	DAT	≥1:8	5
(61)	Bulgaria	37	1 (2.7)	MPA	–	5
(62)	France	195	9 (4.61)	MAT	≥1:25	6
(63)	Norway	361	0	DAT	–	6
(64)	UK	190	2 (1.05)	Unknown	–	6
(65)	Switzerland	615	26 (4.23)	ELISA	–	6
(66)	Cyprus	494	138 (27.94)	IFAT	≥1:240	6
(67)	Italy	74	44 (59.46)	MAT	≥1:20	9
(68)	Serbia	80	22 (27.5)	MAT	≥1:25	6
(69)	Sweden	148	0	DAT	–	6
(70)	France	710	29 (4.08)	MAT	≥1:6	7
(71)	France	77	6 (7.79)	MAT	≥1:6	6
(72)	Italy	128	37 (28.91)	Indirect ELISA	–	6
(73)	Czech Republic	229	6 (2.62)	LAT	–	6
(74)	France	130	4 (3.08)	MAT	–	6
**North America**						
(75)	USA	52	10 (19.23)	SFDT	≥1:32	5
(76)	Canada	21	0	SFDT	≥1:16	5
(77)	USA	559	14 (2.5)	IHAT	–	6
(78)	Canada	116	6 (5.17)	SFDT	≥1:16	6
(79)	Costa Rica	123	12 (9.8)	SFDT	–	6
(80)	USA	681	21 (3.08)	IHAT	≥1:64	6
(81)	USA	109	54 (49.5)	IFAT	–	6
(82)	USA	618	2 (0.32)	MAT	≥1:32	6
(83)	USA	28	2 (7.14)	ILAT	≥1:32	5
(84)	USA	104	11 (10.58)	SFDT	≥1:8	6
(85)	USA	1,399	35 (2.5)	MAT	≥1:25	6
(86)	Panama	797	54 (6.78)	DAT	–	6
(87)	USA	545	51 (9.3)	MAT	≥1:25	6
(88)	Canada	151	16 (10.6)	MAT	≥1:25	6
(89)	USA	93	3 (3.23)	MAT	≥1:25	6
(90)	USA	756	6 (0.8)	MAT	≥1:25	6
(91)	USA	47	4 (8.51)	MAT	≥1:10	5
(92)	USA	62	6 (9.68)	MAT	≥1:25	5
(93)	Grenada	238	2 (0.84)	MAT	≥1:40	6
(13)	USA	447	85 (19.02)	IFAT	≥1:80	7
(13)	USA	76	3 (3.95)	LAT	≥1:32	7
(94)	Mexico	445	6 (1.35)	MAT	≥1:25	6
(95)	USA	35	5 (14.28)	IFAT	≥1:25	8
(96)	USA	66	3 (4.55)	MAT	≥1:25	5
(97)	Mexico	60	7 (11.67)	Indirect ELISA	–	5
(98)	USA	124	13 (10.48)	IFAT	≥1:25	7
(99)	USA	23	1 (4.35)	MAT	≥1:32	5
(100)	Grenada	167	1 (0.6)	MAT	≥1:25	6
**South America**						
(101)	French Guiana	89	24 (26.97)	DAT	>1:40	6
(16)	Brazil	149	63 (42.28)	MAT	≥1:25	6
(102)	French Guiana	127	31 (24.41)	DAT	–	6
(15)	Brazil	64	156 (52)	MAT	≥1:25	6
(103)	Brazil	182	5 (2.75)	MAT	≥1:50	6
(104)	Brazil	26	16 (61.54)	IFAT	≥1:16	8
(105)	Brazil	43	0	MAT	–	5
(106)	Brazil	137	32 (23.36)	MAT	≥1:25	6
(107)	Brazil	34	13 (38.24)	MAT	≥1:25	5
(108)	Brazil	174	10 (5.75)	MAT	≥1:25	9
(109)	Brazil	31	5 (16.13)	IFAT	≥1:16	6
(110)	Argentina	176	49 (27.84)	MAT	≥1:32	6
(111)	Brazil	151	13 (8.61)	MAT	≥1:25	6
(112)	Brazil	170	17 (10)	IFAT	≥1:16	8
(113)	Brazil	182	9 (4.95)	IFAT	≥1:16	8
(114)	Brazil	63	2 (3.17)	MAT	≥1:25	8
(115)	Brazil	178	10 (5.62)	IFAT	≥1:16	6
(116)	Brazil	31	5 (16.13)	MAT	≥1:16	5
(117)	Brazil	46	7 (15.22)	MAT	≥1:25	5
(118)	Brazil	101	3 (2.97)	MAT	≥1:25	8

*SFDT, Sabin-Feldman dye test; LAT, latex agglutination test; MAT, modified agglutination test; IFAT, indirect fluorescent antibody test; IHAT, indirect hemagglutination test; ELISA, enzyme-linked immunosorbent assay; DAT, direct agglutination test; ICT, immunochromatographic assay; ILAT, indirect latex agglutination test; EIA, enzyme immunoassay; CFT, complement fixation test; MPA, microprecipitation method in agar gel*.

**Figure 2:**
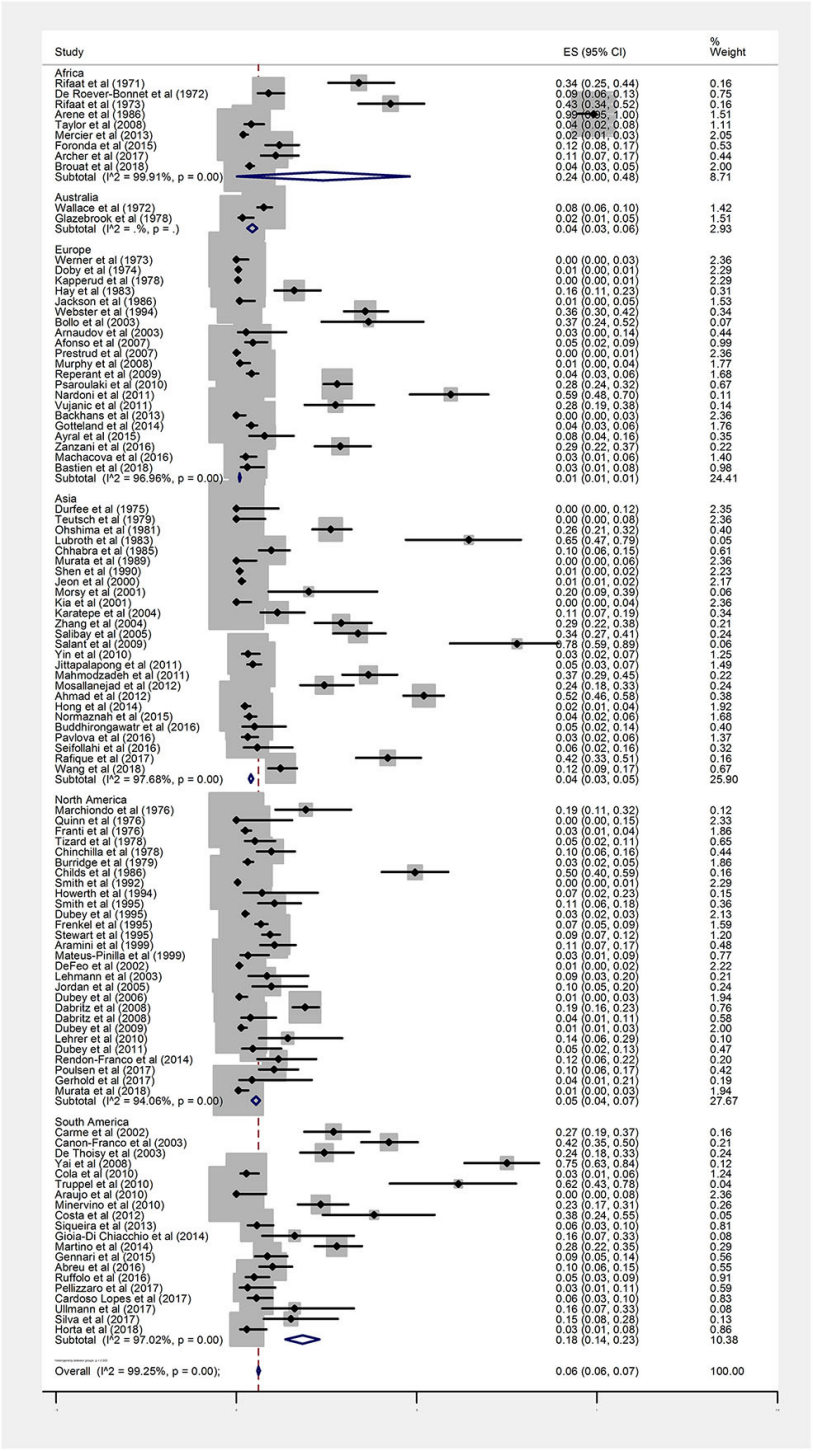
Forest plot diagram for seroprevalence of anti*-Toxoplasma* IgG antibodies in rodents (ES, effect size; CI, confidence interval).

The overall seroprevalence of anti-*Toxoplasma* IgG antibodies in rodents based on the random effect model was calculated at 6% (95%, CI = 6–7%). I-squared statistics indicated a high heterogeneity among the studies (*I*^2^ = 99.25%, *P* < 0.001). Figure 2 demonstrates a forest plot diagram of the current research. In the present analysis (by continental regions), the highest seroprevalence was evaluated in Africa and South America with the amounts of 24% (95% CI = 0–48%) and 18% (95% CI = 14–23%), respectively.

The seroprevalence in North America, Australia, and Asia was measured at 5% (95% CI = 4–7%), 4% (95% CI = 3–6%), and 4% (95% CI = 3–5%), respectively. The Europe had the lowest seroprevalence with 1% (95% CI = 1–1%).

**Figure 3:**
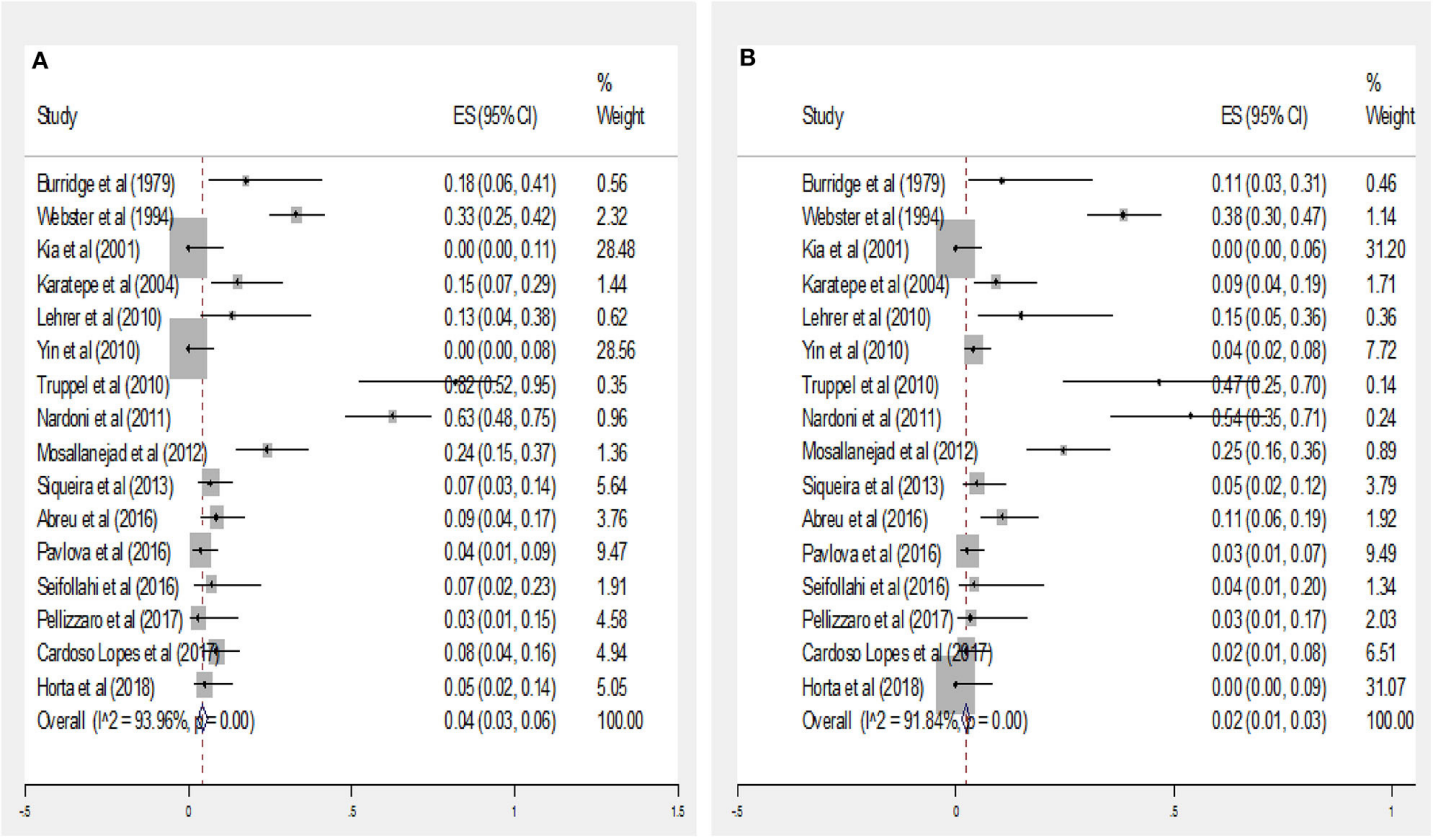
Forest plot diagram for seroprevalence of anti*-Toxoplasma* IgG antibodies in male **(A)** and Female **(B)** rodents (ES, effect size; CI, confidence interval).

The subgroup data analysis of 16 documents describing values of the seroprevalence parasite by gender manifested that the pooled seropositivity value in male and female rodents was 4% (95% CI = 3–6%) and 2% (95% CI = 1–3%), respectively (Figures 3A,B). There was no statistically significant difference between these two groups due to the overlap of CI (*P* > 0.05).

In a subgroup analysis based on serological methods, the highest seroprevalence was found by IFAT 18% (95% CI = 13–23%), followed by LAT, SFDT, ELISA, MAT, IHAT, and DAT with the rate of 15% (95% CI = 9–22%), 14% (95% CI = 12–17%), 11% (95% CI = 7–15%), 8% (95% CI = 7–9%), 3% (95% CI = 1–6%), and 0% (95% CI = 0–1%), respectively. Other serological methods [e.g., indirect latex agglutination test (ILAT), immunochromatographic assay (ICT), enzyme immunoassay (EIA), complement fixation test (CFT), and microprecipitation method in agar gel (MPA)] showed the infection rate of 8% (95% CI = 4–11%; Table 2).

**Table 2:** Sub-group analysis of the seroprevalence of *T. gondii* based on geographical regions, serological methods and gender of rodents.

**Sub-groups**	**Number of studies**	**Total samples**	**Positive samples**	**Pooled Prevalence (95% CI)**	**Weight (%)**	**Heterogeneity**			
						**χ^2^**	**df**	***P-*value**	***I*^2^(%)**
**Geographical regions**									
Africa	9	3,058	311	24% (0–48%)	8.71	9028.79	8	<0.001	99.91%
Australia	2	837	53	4% (3–6%)	2.93	NA	1	NA	NA
Europe	21	5,991	454	1% (1–1%)	24.41	658.07	20	<0.001	96.96%
Asia	26	6,239	650	4% (3–5%)	25.90	1078.01	25	<0.001	97.68%
North America	28	7,942	433	5% (4–7%)	27.67	454.71	27	<0.001	94.06%
South America	20	2,154	362	18% (14–23%)	10.38	637.44	19	<0.001	97.02%
**Serological methods**									
SFDT	17	4,019	338	14% (12–17%)	19.39	11098.14	16	<0.001	99.86%
Other	9	1,142	134	8% (4–11%)	8.55	189.40	8	<0.001	95.78%
IHAT	5	2,406	67	3% (1–6%)	6.61	30.45	4	<0.001	86.86%
LAT	9	1,842	321	15% (9–22%)	8.33	548.79	8	<0.001	98.54%
IFAT	14	2,628	413	18% (13–23%)	9.14	610.10	13	<0.001	97.87%
MAT	39	9,878	658	8% (7–9%)	34.54	1019.74	38	<0.001	96.27%
DAT	7	1,720	177	0% (0–1%)	7.02	235.44	6	<0.001	97.45%
ELISA	6	2,586	155	11% (7–15%)	6.41	135.32	5	<0.001	96.31%
**Gender**									
Male	16	859	131	4% (3–6%)	NA	248.21	15	<0.001	93.96%
Female	16	1,065	124	2% (1–3%)	NA	183.83	15	<0.001	91.84%

*df, degrees of freedom; NA, not available (parameter not provided)*.

**Figure 4:**
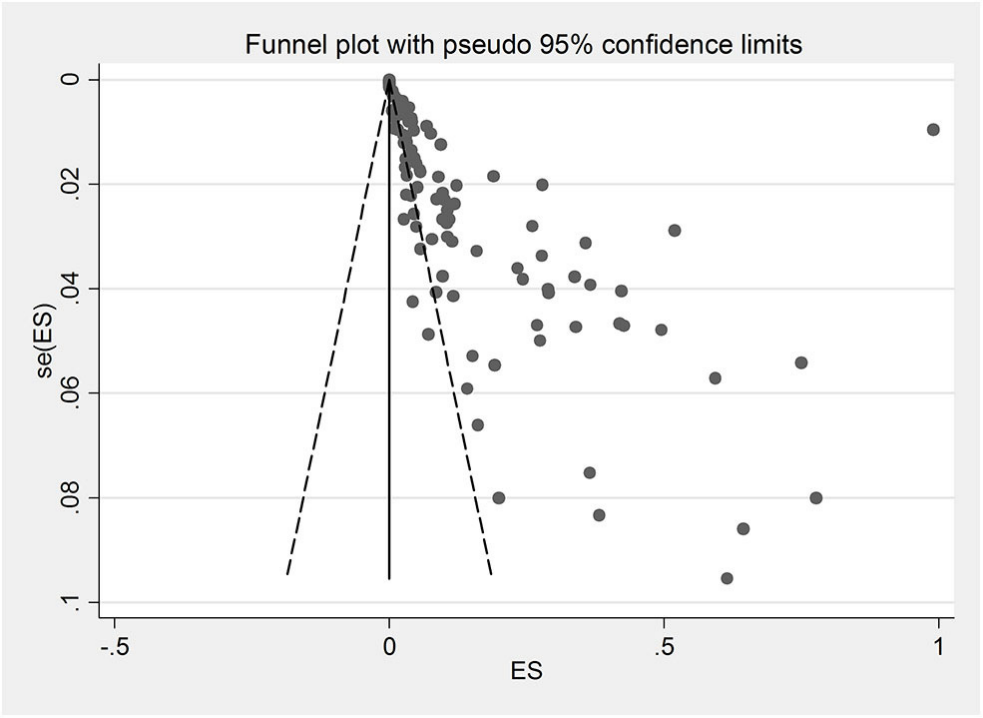
Funnel plot for detecting publication bias.

Due to the lack of adequate data on the rodents' age, subgroup analysis was not performed. The results of Egger's regression test indicated that publication bias was statistically significant (Egger bias: 5.650, *P* < 0.001). Figure 4 shows the Funnel plot for this purpose.

To detect the sources of heterogeneity among different studies, meta-regression analysis was applied based on serological methods and continental regions, the results showed that the illustrated values were not statistically significant (*P* = 0.480 and *P* = 0.295, respectively). The sensitivity analysis tool demonstrated that the effect of three studies on the overall effect size was significant (22, 63, 69).

## Discussion

Toxoplasmosis, one of the most common infections in humans, is important both medically and economically. It causes many serious consequences in humans and animals with economic importance (e.g., livestock). It has been recorded that infection leads to abortions in many mammals (e.g., rodents, and livestock), could inhibit species recovery, and cause economic losses (5, 10, 119).

Rodents, as intermediate and reservoir hosts of this protozoan, can be contaminated and maintain the parasite in the form of cysts in their bodies, demonstrating an infection source for their offsprings, predators (particularly felids), and other animals (If rodents' bodies are accidentally eaten by them) (6, 11, 14). It has been shown animals such as livestock and pigs that are economically important, may accidentally or intentionally eat live small rodents or their carcasses and thus can get infection via digesting tissue cysts without the intervention of definitive hosts (11, 59).

By establishing the infection transmission cycle and consequently environment contamination by released oocysts from cats, rodents, especially species that live close to humans such as house mice, lead to increasing the risk of human exposure to the parasite (14). The rodent capybara that is used by humans in many countries of South and Central America, may be a potential source of infection for humans if its meat contains parasitic cysts and is consumed insufficiently cooked (15, 16).

Also, the consumption of rats as food by some populations may increase the risk of direct transmission from these rodents to humans, when eating, handling or preparing infected rats before cooking (3). Hence, people who consume rodents' meat should follow the principles of hygiene during meat preparation and cook the meat properly.

In the sylvatic transmission cycle in forest habitats, rodents as important wildlife intermediate and reservoir host of *T. gondii*, with maintaining parasite and its transmission cycle in these ecosystems may lead to increasing the probability of infection of wild felids and other animals, especially if they are the main prey (3).

Transmission of the parasite from wildlife to human habitats may rarely and accidentally occur by moving infected rodents and other parasite hosts. The accidental transportation of infected rodents from one region to another by human trade activities and other pathways causes strains to be transmitted internationally and sometimes new strains are introduced in the region (120).

Therefore, these mammals play a substantial role in the transmission of the infection to felids and most animals, as well as the dissemination of the infection in the environment and the risk of human infection. Consequently, comprehensive studies are required to reveal the status of toxoplasmosis in rodents and to better develop control measures and strategies. Hence, conducting further studies could help to reduce the infection rate in these mammals, decrease environmental contamination, and mitigate the risk of infection transmission. In order to achieve these goals, the current study was carried out to investigate the seroprevalence of *T. gondii* in rodents.

The present extensive study was the first systematic review to concentrate on the worldwide seroprevalence of toxoplasmosis in rodents, by screening scientific studies published from 1970 to 2018. In this attempt, the overall seroprevalence of anti-*T. gondii* IgG antibodies was calculated at 6% (95% CI = 6–7%), with the highest amount in Africa (24%), South America (18%), and the lowest amount in Europe (1%).

Our results illustrated a large variation in the seroprevalence of infection in various studies, ranging from 0 to 100%. In general, these variations were observed in different studies and geographical areas and were influenced by numerous factors, including abundance of definitive and intermediate hosts, distinct ecologic patterns, the sensitivity of used methods, variability in vertical transmission or susceptibility to infection between species, differences in climate conditions, and environmental factors (e.g., mud and water) affecting the sporulation and survival of oocysts (41, 70, 98). Depending on the situation, some of these factors had a more substantial role in the variation of the seroprevalence of infection than others. Therefore, it may be difficult to compare the results of different studies due to differences in important factors such as serological tests (with the variable sensitivity, specificity, and cut-off), rodent species, etc. The rodents resembling other mammals were contaminated with *T. gondii* through eating the infective oocysts residing in water, soil, and food. Also, ingestion of meat infected with parasite tissue cysts (via cannibalism), digesting earthworms as paratenic hosts of the pathogen, or congenital transmission lead to contamination of the rodents with *T. gondii*. The high level of congenital transmission recorded among some rodent species could affect the prevalence levels (64, 121). For example, congenital transmission occurs high in the wild rat populations, thus it can be an important route in parasite transmission and maintenance and lead to high prevalence levels in this species, regardless of environmental contamination (59).

The contamination level of the soil is different according to the region, depending on the densities of felids as definitive hosts, which excrete oocysts into the environment. In fact, rodents living in an environment with fewer cats, are less exposed to oocysts (62, 121). As high infection rates have been reported in rodents living in rural areas such as mice (59%) and rats (70%), because they are more in contact with cats and their feces (48).

The humid and warm climate is a suitable condition for the survival and dissemination of oocysts; therefore, areas with these climates show a high level of infection. In addition, water and damp soil can support the stability of oocysts for longer periods (43, 108). The oocysts are able to survive in moist soil for up to a year and low humidity and high temperatures can kill them (76, 118). Rodents such as muskrats that swim in water or semi-aquatic species (capybaras) show higher infection rates (ranging from 17 to 60%) than those that are less exposed to water environments (13). The seasonal variations of climate also affect the infection rate as it increases in the wet seasons than the dry seasons (98).

The estimation of seroprevalence infection may not reflect the actual amount of infected individuals, because, contrary to the resistance of some species to infection, others may be more susceptible and show more casualties. Therefore, the casualties are not included in the study and the reported amount will not be actual (121).

The antibody production is affected by various factors such as parasite genotype, infection persistence, and host age, etc., also the duration of immunity may vary and in some species, the antibody produced is reduced after a short time and becomes unrecognizable (76). On the other hand, some congenitally infected rats and mice do not develop antibodies while harboring parasites in their tissues (100). The used diagnostic techniques were very important, because of the low specificity and sensitivity of serological methods and usage of an improper cut-off value, may over/underestimate pathogen prevalence by false negative or positive results (121). Many researchers have shown that the prevalence of infection in rodents might be estimated less or more than the actual value when relying on serology, compared to other valid techniques, such as bioassay, as the gold standard for the diagnosis of *T. gondii* infection, or Polymerase chain reaction (PCR) (10, 14, 64, 69, 70). Regarding, the use of the serology technique along with bioassay and PCR could provide a more accurate estimation of the infection rate in rodents. According to the subgroup analysis of serological methods, the highest seroprevalence was detected by IFAT, followed by LAT, SFDT, ELISA, and MAT. Studies that used LAT, reported the lowest seroprevalence. These differences in the estimation of the prevalence may be due to the variable specificity, sensitivity, and cut-off of the used serological tests. Based on our findings, the pooled global seroprevalence of antibodies against *T. gondii* among rodents was relatively low. Although the infection levels in cats will be affected by the contamination levels in their consumed prey, a low infection rate among rodents may account for a high infection rate in cats since these animals may consume hundreds of rodents throughout their living (56). In fact, it is possible to relate the seroprevalence rates in felids and the number of rodents consumed, which varies according to the prey abundance, season, and local conditions (76, 122).

Considering the different prey availability according to the habitat and unequal effect of prey species on the infection risk for predators, examining both the predominant prey of felids and the prevalence of infection in them can help to better predict the *T. gondii* infection risk of felids in specific habitats (121).

Moreover, *T. gondii* has been demonstrated to be responsible for change of behavior patterns among rodents (e.g., increased attraction to felids urine, losing their innate fear of cats, and causing neurological impairment), which increases the risk of predation of the infected rodents and lead to infection transmission to the felids (70, 95, 98). Given the above, a low number of rodents infected with toxoplasmosis may lead to high transmission in felids and other predators depending on the situation.

Publication bias was statistically significant in the selected studies, probably for reasons, such as sample size, sampling procedure, and methodology.

In our study, it was concluded that the high seroprevalence of infection among rodents in Africa and South America was due to climate conditions and other aforementioned factors indicating an increased risk of infection transmission to felids, humans, and other animals. Therefore, effective control measures and strategies should be implemented in order to reduce the infection rates among rodents in these regions.

Data analysis of the few studies reporting infection rates by gender in rodents suggested that the prevalence of *T. gondii* antibodies did not differ between the genders with 4% in males and 2% in females, suggesting that both genders are almost equally exposed to this parasite. In some species of rodents such as rats, males have larger home ranges than females and thus a greater chance for acquiring infection (59).

Due to the lack of adequate data on rodents' age in the selected studies, subgroup analysis of age groups was not performed. In general, because of spending more time in the environment and the increased risk of exposure to parasites, the seropositive rate of *T. gondii* has been expected to be higher in aged animals than in younger ones, as shown in numerous animal species and humans (2, 123–126).

Cannibalism, one of the routes of infection transmission in rodents that is observed in some species, is more common in males and older animals than in females and younger ones, that can affect the burden of infection (59). Hence, specific feeding, foraging or social behaviors observed in rodents that vary from one species to another can determine the extent of exposure to the parasite and the differences in prevalences related to the sex and maturity in any species (3).

## Conclusions

In conclusion, the present study revealed a relatively low level of *T. gondii* seroprevalence in rodents; however, if they were the main food source for their predators, they would cause high transmission and subsequently increase environmental contamination and the risk of infection transmission to humans and other animals.

Consequently, effective control measures and strategies are needed to reduce the infection rate in these mammals. Further studies are required to use the serology technique along with bioassay and PCR to provide a more accurate estimation of the infection rate in these animals.

## Data Availability Statement

The original contributions presented in the study are included in the article/supplementary material, further inquiries can be directed to the corresponding author/s.

## Author Contributions

AD and ShS contributed to the design of the study. TG and MMon conducted the systematic review of the literature and extracted data. MMoo performed all statistical analyses, data interpretation, and drafted the manuscript. TG contributed to the interpretation of data and writing of the first draft. AD supervised the study. All authors read and approved the final manuscript.

## Conflict of Interest

The authors declare that the research was conducted in the absence of any commercial or financial relationships that could be construed as a potential conflict of interest.

## Abbreviations

CI: confidence interval
PRISMA: Preferred Reporting Items for Systematic Review and Meta-Analysis
JBI: Joanna Briggs Institute
IgG: Immunoglobulin G
MAT: modified agglutination test
SFDT: Sabin-Feldman dye test
IFAT: indirect fluorescent antibody test
LAT: latex agglutination test
DAT: direct agglutination test
ELISA: enzyme-linked immunosorbent assay
IHAT: indirect hemagglutination test
ILAT: indirect latex agglutination test
ICT: immunochromatographic assay
EIA: enzyme immunoassay
CFT: complement fixation test
MPA: microprecipitation method in agar gel
PCR: Polymerase chain reaction.
